# Threatment Strategies for Recurrent Hepatocellular Carcinoma Patients: Ablation and its Combination Patterns

**DOI:** 10.7150/jca.93885

**Published:** 2024-02-25

**Authors:** Ya'ning Zhao, Jun Bai, Xi Wang, Yaoren Zhang, Xiaohong Yan, Jun'an Qi, Xueyan Xia, Yuansong Feng, Baojun Duan

**Affiliations:** 1 Department of Medical Oncology of Baoji Central Hospital, Baoji 721008, Shaanxi Province, China.; 2 Department of Medical Oncology of Shaanxi Provincial People's Hospital, Xi'an 710068, Shaanxi Province, China.; 3 Department of Ultrasonography of Baoji Central Hospital, Baoji 721008, Shaanxi Province, China.; 4 Department of Hepatobiliary Surgery of Baoji Central Hospital, Baoji 721008, Shaanxi Province, China.

**Keywords:** Ablation, HCC, RFA, MWA

## Abstract

With the development of guidance technology and ablation equipment, ablative procedures have emerged as important loco-regional alternatives to surgical resection for recurrent hepatocellular carcinoma (rHCC) patients. Currently, ablation modalities used in clinical practice mainly include radiofrequency ablation (RFA), microwave ablation (MWA), laser ablation (LA), cryoablation (CRA), high-intensity focused ultrasound (HIFU), and irreversible electroporation (IRE). Accumulated comparative data of ablation versus surgical resection reveal noninferior responses and outcomes but superior adverse effects. Moreover, studies demonstrate that ablation may serve as an excellent procedure for rHCC given its exact minimal invasiveness and immune modulation. We focus on the current status of ablation in clinical practice for rHCC and discuss new research in the field, including ablation combined with these other modalities, such as targeted therapy and immunotherapy.

## Introduction

The recurrence rate after liver transplantation and hepatectomy is 11-18% and 70%, while liver-only recurrence is approximately 33% and 90% respectively 1-4. These data show that intrahepatic recurrence is the most common type among all types of recurrence 5.

At the time of recurrence, the prognosis of rHCC patients depend on age, platelet count, the AFP value, the number of visually visible recurrent tumors, tumors size, gross vascular invasion, Child‒Pugh classification, coexisting liver disease and severity 6,7. Thus, it is important to thoroughly and carefully assess liver function and portal hypertension status before administering anti-tumor therapy. National Comprehensive Cancer Network (NCCN) recommends a multidisciplinary assessment analysis of patients, including liver function, staging, and Eastern Cooperative Oncology Group (ECOG) score, and discusses antiviral and comprehensive management of coexisting liver disease 8. At present, assessment protocols for liver function, tumor staging, and first-line treatment decisions in patients with rHCC are lacking, but treatment strategies from the Barcelona Clinical Liver Cancer (BCLC) and the Japanese Society of Liver Diseases can be extrapolated and used as treatment guidelines 9,10.

Currently, there is no consensus on a standardized treatment strategy for patients with rHCC, however, different treatment modalities can affect their survival. Patients with local recurrence in the liver may be considered for repeat hepatectomy, liver transplantation or loco-regional therapy (LRT), which including ablation, arterially directed therapy, and irradiation etc. Hepatectomy is only indicated for patients with Child‒Pugh grade A or partial Child‒Pugh grade B cirrhosis and no evidence of portal hypertension. When repeatable hepatectomy is not possible, liver transplantation is an effective treatment for rHCC provided that the criteria for transplantation are met and that the patient's physical status score indicates that he or she can tolerate the transplantation procedure. In general, both repeat hepatectomy and transplantation have similar overall survival rates, but the transplantation procedure is mainly limited by the organ source 11. LRT is choice for patients in good physical status with tumor lesions limited to the liver and preserved liver function or as a bridging treatment modality for patients awaiting liver transplantation. In clinical practice, LRT also may be considered in patients with portal vein cancer thrombosis and limited extrahepatic disease. As one kind of LRT, findings from numerous studies with other solid tumor types help us to understand the properties of ablation on HCC, and with the development of imaging guidance and ablation devices, ablation has become an important therapeutic technique for HCC. Moreover, for patients with evidence of recurrent extrahepatic disease, ablation combined with systemic therapy or participation in clinical trials may be required 1.

## Ablation- An effective and minimally invasive LRT

Ablation leads to localized tumor cell necrosis or apoptosis, induces destruction of specific areas of the lesion and minimizes damage to surrounding normal tissues and structures. In order to prevent and avoid local tumor recurrence, a safety zone (at least 0.5cm) around the ablation-treated lesion is required 12,13. Findings from numerous studies with other solid tumor types help us to understand the properties of ablation on HCC. With the development of imaging guidance and ablation devices, ablation has become an important therapeutic technique for HCC patients.

In clinical practice currently, when the term "ablation" is used, it mainly refers to RFA or MWA, of which RFA has been established as the standard treatment for HCC for decades. Nonthermal ablation techniques, including anhydrous ethanol or acetic acid injection, CRA and IRE, may be considered for lesions that cannot be thermally ablated given their proximity to vital ducts (vessels or bile ducts) or organs (diaphragm or gastrointestinal tract) 8. Notably, other ablation techniques, such as CRA and IRE, have not been included in published guidelines due to insufficient evidence.

The indications for ablation depend on factors such as tumor size, type, stage, patient ECOG score, liver function and comorbidities. In appropriately selected (<3-5cm) patients, ablative therapy has resulted in 5-year overall survival rates of 26% to 71% and can be used in patients with combined portal hypertension 14. For early intrahepatic recurrence, repeat hepatic resection and ablation have similar efficiency rates as Transarterial chemoembolization (TACE), and repeat hepatic resection and ablation are the treatment of choice for patients with advanced recurrence after radical resection for HCC meeting the Milan criteria, whereas ablation therapy can also be used for bridging or descending prior to surgery or transplantation 15. The overall survival of patients with rHCC after repeat hepatectomy and ablation is similar to that of patients with primary tumor resection 16. However, compared to repeat hepatectomy and salvage liver transplantation, ablation has fewer complications, lower incidence of postoperative liver dysfunction and can be repeated because it is minimally invasive 17,18.

Fusion imaging and enhanced ultrasound have significantly improved the technical success of ablation, whereas multi-electrode-based "no-touch" ablation techniques produce a larger ablation area and significantly reduce the local recurrence rate 19. Moreover, ablation combined with or without TACE may also be considered for patients with BCLC C in the context of multidisciplinary discussions and when no other treatment options are available 20.

## Chemical ablation- May be an option in certain circumstances

Chemical ablation could deactive lesions <2cm effectively, this treatment is safer than thermal ablation when treating lesions at risk sites (adjacent to vital ducts or organs). In addition, chemical ablation is rapid and relatively cheaper than other energy-dependent ablation techniques 20. For tumors larger than 2cm, multiple intratumoral injections must be repeated to ensure adequate coverage of the entire tumor lesion and safe margins. Factors that are associated with recurrence after chemical ablation mainly include ineffective control of local diffusion of the injected reagent, uneven distribution of the injected drug in larger tumors, and difficulty in obtaining adequate treatment margins.

Data from 3510 HCC patients (≤ 5 cm) in the surveillance epidemiology and end results (SEER) database showed that the median overall survival (mOS) and median cancer-specific survival (mCSS) of PEI-treated patients were not significantly different from those of RFA-treated patients before or after propensity score matching (PSM). Subgroup analysis showed no significant difference in mOS between PEI and RFA in patients regardless of tumor size and American Joint Committee on Cancer (AJCC) staging. Multivariate regression analysis showed that PEI did not increase the risk of all-cause mortality or the risk of cancer-specific mortality after PSM. For patients with a single HCC, RFA remains the better option, however, for patients who cannot be treated with RFA, PEI may represent a good option 21.

## RFA- Major procedure for the treatment of rHCC

Percutaneous "no-touch" RFA is an effective modality for treating ≤ 5 cm HCC with an overall local tumor progression (LTP) rate of 6%, which is lower than that of conventional RFA (intra-tumoral puncture) 22. Both RFA and repeat surgical resection (RHR) are reasonable treatment options for rHCC, but it is uncertain which approach is better. A stratified comparison of the outcomes of RHR and ablation based on the number and size of recurrent tumors may better elucidate the best treatment approach. In this article, we focus on the following aspects: 1. Survival: Several studies showed that the long-term (3-year and 5-year) overall survival (OS) and disease-free survival (DFS) of RHR are significantly higher than those of RFA, but there is no significant difference in the short-term (1-year) OS and DFS. In addition, pooled results showed that RHR has higher OS, DFS and progression-free survival (PFS) in the treatment of rHCC 23-25. However, other data demonstrate similar OS and PFS at 1, 3 and 5 years for RFA and RHR 26,27. 2. Complications: Compared with RHR, RFA has a lower postoperative complication rate and shorter hospitalization time. When survival is the primary goal, RHR should be the first choice for rHCC. However, the advantage of lower major complications may make RFA an alternative treatment option for specific patients 23,24,26,27. 3. Lesion size and number: For larger lesions (< 5 cm, > 3 cm), RHR may be associated with better local disease control. For small lesions (≤ 3 cm), RFA and RHR have similar effects 24,26,28. Moreover, RFA is more effective and safer than RHR in patients with 2 or 3 tumor lesions 27. 4. Milan criteria: In the subgroup meeting the Milan criteria, RHR yielded similar 1, 3, and 5 year OS and 1 year DFS values as RFA but better 3 and 5-year DFS values. RFA is the first choice for rHCC meeting the Milan criteria. When it does not meet the Milan criteria, minimally invasive treatment should not be performed at the cost of survival, and RHR should be the first choice 25. Therefore, RHR is superior to RFA in improving the survival of rHCC. Compared with RFA, RHR is associated with longer recurrence free survival, which provides survival advantages for rHCC, especially for patients who relapse within 2 years and patients whose primary tumor burden exceeds Milan criteria. In addition, it has been suggested that the vessels encapsulating tumor clusters model could be potentially used to select patients for whom RHR is appropriate 29-31.

In general, RHR and RFA have their own advantages and disadvantages for the treatment of rHCC, and carefully constructed, randomized and multicentre trials are needed to determine whether real differences exist between two modalities.

Notably, RFA suffers from the same problem of relapse as the other modalities. Recent research found that Methyltransferase 1 (METTL1) plays a key role in regulating the immunosuppressive microenvironment, and blocking the METTL1-TGF-β2-PMN-MDSC axis may prevent HCC relapse after RFA treatment by restoring antitumor immunity, which warrants further investigation 32.

## MWA- Higher thermal efficiency than RFA

Compared with RFA, MWA has the following advantages: higher energy, ablation of larger lesions, less susceptible to the "heat sink" effect, and simultaneous ablation of multiple lesions. However, no significant difference in clinical efficacy is noted between MWA and RFA 20,33,34.

Regardless of lesion size and vascular adjacency, MWA has a lower local progression rate than unipolar RFA, but no significant difference in the risk of distant tumor progression is noted 35. Compared with RFA, MWA is better suited for HCC meeting Milan criteria, whereas both RFA and MWA have similar efficacy for lesions ≤ 3 cm 36. MWA ablation is better for subcapsular lesions, but no significant differences in complications between these two modalities were noted 37.

An analysis including 4 randomized controlled trials and 10 cohort studies showed that there was no significant difference between MWA and RFA for percutaneous ablation in terms of complete ablation (CA), local recurrence (LR), DFS, OS, and major complication rates. For laparoscopic ablation, MWA had a lower LR, but no significant differences in CA, DFS, OS or major complications were noted between RFA and MWA 34,38. Another meta-analysis involving 33 studies, including 4589 patients, showed that MWA had higher CA and lower LTP than RFA in HCC, but no significant differences in OS and major complications were noted 39.

## CRA- Plays a synergistic role with other antitumor therapies

Compared with thermal ablation, CRA is safer for lesions adjacent to (within 1 cm) other organs (such as the gallbladder and gastrointestinal tract) 40. In addition, CRA has analgesic effects, and CRA of liver tumors with pain or metastatic lesions in the pleura and chest wall can reduce pain for 5 to 8 weeks after the procedure 41. However, unlike thermal ablation (RFA and MWA), CRA cannot ablate the puncture needle path to play a haemostatic role, thus, it is necessary to insert a haemostatic sponge into the puncture needle path through a coaxial needle to stop bleeding 42,43.

Although CRA is a very promising modality, it does not offer significant advantages compared with other thermal ablation modalities for the treatment of HCC. A retrospective study based on the SEER database showed that before PSM, the mOS and mCSS of the RFA group were slightly longer than those of the CRA group. In the subgroup analysis, the mOS and mCSS of patients with tumor sizes < 3 cm, 3-5 cm and > 5 cm who received RFA treatment were longer than those who received CRA, but the difference was not significant. Similar results were also observed in AJCC stage I and II patients. After PSM, the mOS and mCSS of the RFA group were slightly higher than those of the CRA group, but no significant difference was observed. Univariate and multivariate analyses showed that CRA treatment was not an adverse factor for OS and CSS before and after PSM. For single HCC patients without lymph node invasion or distant metastasis, CRA is not inferior to RFA 44.

Multifunctional nanomaterials can improve the therapeutic efficacy of CRA by influencing ice formation and freeze-induced cell death, more precise formulation of the CRA area, and visualization of the ablation zone. Taking advantage of these characteristics, combined CRA therapy based on nanomaterials can improve the therapeutic effect of traditional CRA 42.

Of note, cell death caused by CRA is more likely to induce inflammatory responses and antitumor antigen release, suggesting that CRA seems to better synergize with immunotherapy 45.

## LA- Feasible method for the treatment of HCC

Multicentre retrospective analysis found that for early HCC patients (single nodule ≤ 4 cm or three nodules ≤ 3 cm) with cirrhosis receiving LA treatment, the complete remission rate was 78%, the mOS was 47 months, and the 3 and 5-year cumulative survival rates were 61% and 34%, respectively. Multivariate analysis showed that serum albumin levels greater than 3.5 g/dl, complete tumor ablation, and age less than 73 years were independent predictors of survival. In patients with tumors ≤ 2 cm, the 5-year OS was 60%, and the mOS was 63 months 46.

Orlacchio et al. compared the efficacy of LA and RFA for the treatment of HCC (≤ 4 cm) with liver cirrhosis. RFA is more effective for lesions ≥ 21 mm, whereas LA is more effective for lesions ≤ 20 mm. LA has a lower incidence of complications compared with RFA 47.

A retrospective study compared the efficacy and safety of MWA and LA for the treatment of HCC. The LTP and intrahepatic distant recurrence rate were 6% and 46% in the MWA group, and 3.8% and 64.2% in the LA group. The 1, 3 and 5-year OS rates of the MWA group were 94.3%, 65.4% and 49.1%, and those of the LA group were 96.2%, 54.7% and 30.2%. The DFS rates at 1, 2 and 3 years were 45.9%, 30.6% and 24.8% in the MWA group, and 54.7%, 30.2% and 17%, in the LA group. The initial CA rates of the MWA group and LA group were 97.7% and 98.7%, and the total complication rates of the two groups were 2.9% and 7.9%, respectively. Based on these results, MWA is recommended over LA given that the patients in the MWA group had higher survival rates 48.

Chai et al. investigated the effect of LA in the porta-caval area in HCC and found that all patients achieved technical success and CA for the first time without serious complications. The LTP rates at 6 and 12 months were 0% and 10%, respectively, and the distant tumor recurrence rate was 20%. They concluded that LA is safe and feasible in the treatment of HCC in the portacaval area 49.

## HIFU- Associated with challenges in the treatment of HCC

Compared with RFA, HIFU has the following advantages: 1. Avoids targeted tumor puncturing; 2. Tumor seeding along the needle tract will not occurs, which is often in RFA therapy for HCC 50.

A retrospective study on the feasibility and safety of HIFU in the treatment of HCC and metastatic liver cancer showed that the ORR and DCR of HCC and metastatic liver cancer patients were 71.8% and 81.2% and 63.7% and 83.2%, respectively. Compared with baseline, AFP and visual analogue scale levels significantly decreased after HIFU. The OS rates of the HCC and metastatic liver cancer cohorts were 13.0 months and 12.0 month. The 1-year survival rates were 70.69% and 48.00% of the HCC and metastatic liver cancer cohorts, respectively. Serious adverse events rarely occurred. These data suggest that HIFU is an effective and safe treatment for HCC and metastatic liver cancer 51. In addition, HIFU can be used as a bridging therapy for HCC patients who are waiting for organ transplantation 52.

Chan et al. reported the preliminary experience of HIFU for the treatment of rHCC. The DFS rates of the HIFU group and RFA group at 1, 2 and 3 years were 37.0%, 25.9%, and 18.5% and 48.6%, 32.1%, and 26.5%. The OS rates of the HIFU group and RFA group at 1, 2 and 3 years were 96.3%, 81.5%, and 69.8% and 92.1%, 76.1%, and 64.2%, respectively. The complications observed in the HIFU group included skin burns and pleural effusion. No hospital mortality was noted in the HIFU group, whereas 2 deaths occurred in the RFA group. They concluded that HIFU is a promising treatment strategy for rHCC 50.

Due to the effect of respiratory-induced liver motion, partial blocking by the rib cage, and high perfusion/flow, HIFU is associated with challenges in the treatment of liver lesions. Lorton et al. designed a HIFU phased-array transducer dedicated to transcostal hepatic thermoablation. This transducer meets the requirement to perform thermal lesions in deep tissues without the need for rib-sparing methods 53.

## IRE- Effectively ablate lesions in dangerous areas

Unlike thermal ablation, IRE is virtually unaffected by the "heat sink" effect. Furthermore, IRE can effectively ablate lesions in dangerous areas, retain tumor-associated antigens, and activate antitumor immune responses 54,55. However, IRE requires the placement of multiple electrodes in a precise geometric configuration along with general anaesthesia and muscle relaxation, making it costly 56,57.

Nanosecond pulsed electric field (nsPEF) is a new IRE technique uses ultrashort pulses (nanosecond duration) that not only penetrate cell membranes but also act on cell organelles. Preclinical studies have shown that nsPEFs can effectively ablate lesions without damaging vital organs and induce antitumor immune responses. A phase I prospective clinical study of nsPEF in the treatment of HCC has been registered at clinicaltrials.gov (NCT04309747) 58.

A retrospective longitudinal study assessed CT-guided IRE in patients with HCC not eligible for thermal ablation (lesions at the hepatocaval confluence), demonstrating that CA was achieved in all cases. Local and distant recurrence rates of 4.8% and 42.6%, respectively. With the exception of 1 hepatic vein located near the lesion that was temporarily occluded and recovered within 1 month, all postcava remained perfused with PFS of 121 days and mOS of 451.5 days. These results suggest that IRE can safely and effectively treat HCC at the hepatocaval confluence 59.

## Ablation vs. Surgery- Each has its own advantages for selected HCC patients

Surgical treatment has a higher incidence of adverse reactions, longer hospital stay and higher cost, whereas ablation has a higher recurrence rate. However, the higher recurrence rate of ablation does not affect OS 60,61.

Surgical resection is recommended in the absence of significant portal hypertension (portal pressure gradient >10 mmHg) according to BCLC criteria. However, ablation may represent a better option when the patient has a combination of significant portal hypertension or other comorbidities, as surgical resection in these cases is more complex and has higher complications 62. For lesions less than 3 cm, the survival rate of ablation does not seem to be significantly different from that of liver transplantation, but the cost and hospitalization time of ablation are lower. Therefore, ablation is expected to be the preferred recommended treatment for such lesions 63.

Although techniques such as multielectrode ablation or IRE are currently available, these techniques have not been popularized and applied to a greater extent in the treatment of HCC due to the impact of evidence-based medical evidence, economic benefit ratio and equipment availability 8,56.

**Figure 1:**
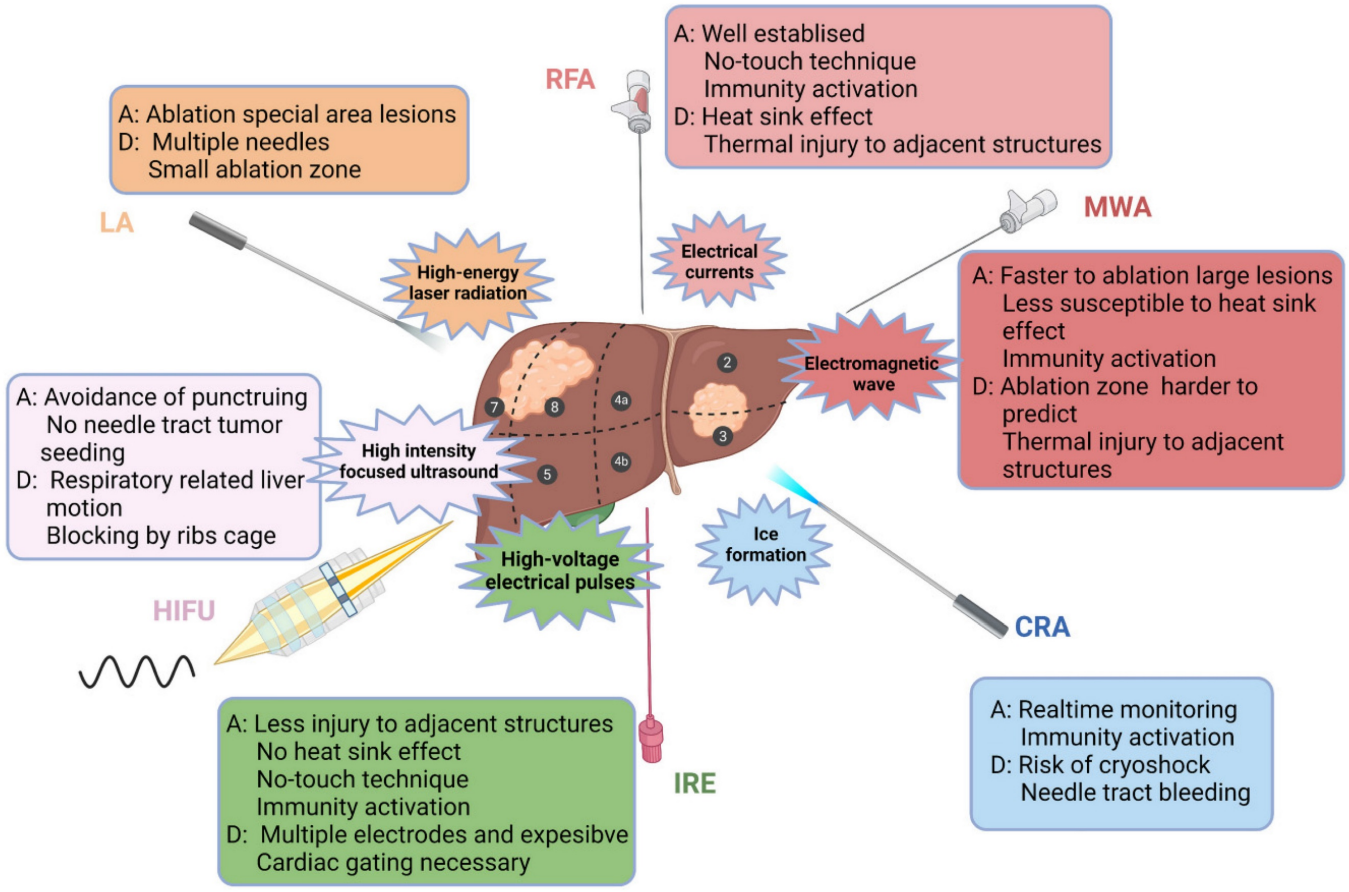
Ablative modalities for the treatment of HCC. Ablative modalities for HCC patients are illustrated according to the mechanism of ablation. The selection of ablative technique depends on the location, size and adjacent relationship with local structures (vasculature, biliary tract, gastrointestinal organs, etc.) of the lesion. RFA, radiofrequency ablation; MWA, microwave ablation; CRA, cryoablation; IRE, irreversible electroporation; HIFU, high-intensity focused ultrasound; LA, laser ablation; A, advantage; D, disadvantage.

Location of the lesion plays an important role in the clinical decision-making regarding the treatment scheme. Ablation has a good therapeutic effect for lesions within the liver parenchyma, but the risk increases if the lesion is adjacent to dangerous sites (large vessels, biliary tract, gastrointestinal tract, and diaphragm) 64. However, with the application of techniques, such as hydrodissection, fusion imaging navigation and nonthermal ablation, ablation for dangerous sites has been increasingly practised. In addition, intraoperative ablation can be considered for larger HCC or adjacent lesions at risk sites 60,61. In summary, ablative modalities for the treatment of HCC are illustrated in Figure 1.

## RFA combined with PEI- Remains controversial regarding clinical benefits

Whether the combination of RFA and PEI provides additional benefit over RFA treatment in patients with HCC remains controversial. A meta-analysis that included 10 studies including 854 patients with histologically confirmed HCC showed a mild improvement in 1, 2, and 3-year OS, 1-year local recurrence-free (LRF) and complete tumor necrosis (CTN) in patients treated with RFA-PEI compared with RFA. However, the incidence of common complications, such as fever, was significantly higher in the RFA-PEI group. This study concluded that RFA-PEI appeared to be superior for HCC patients in terms of OS. However, the current evidence exhibited moderate to significant heterogeneity, and it was difficult to draw a definite conclusion regarding therapeutic management in terms of LRF and CTN 65.

## Ablation combined with TACE- Safe and effective treatment option for large HCC

For HCC patients with unresectable disease, especially for larger tumors and rHCC, ablation combined with TACE has been extensively investigated and applied 66. The concept of combined pattern is mainly based on the following: 1. inhibit TACE-induced neovascularization, thus reducing the risk of tumor recurrence and metastasis 67. 2. effectively expand the area of coagulation necrosis and reduce the rate of LTP 68. A meta-analysis found that TACE combined with RFA is a safe and effective treatment option for HCC, which significantly improves OS and RFS 69.

No significant difference in OS and DFS at 1, 3, and 5 years were noted in the TACE-RFA group compared with the RHR group, but the rate of major complications and length of hospital stay were significantly lower in the TACE-RFA group. After PSM, no significant difference in OS and DFS were noted between the two groups. Among them, AFP and initial tumor microvascular infiltration were important prognostic factors for OS and DFS, respectively. The author suggested that for rHCC patients, in addition to RHR, TACE-RFA is a safe and effective alternative option 70.

In another study comparing the efficacy and safety of TACE combined with MWA (TACE-MWA) with TACE alone for rHCC, it was found that tumor response rates and PFS rates were significantly improved in the TACE-MWA group compared with TACE. No significant differences in the OS rates were noted between the two groups. In addition, no major treatment-related complications were observed in either group, and the number of repeat TACEs was significantly reduced in the combination group compared with the TACE group 71.

Overall, for rHCC, ablation combined with TACE was comparable to RHR in terms of OS and DFS but with fewer major complications and a reduced length of stay. On the other hand, ablation combined with TACE was superior to TACE alone in terms of DCR and OS and had a good safety profile 72.

## Ablation combined with Systematic therapy- Potential to change the therapeutic strategies for HCC

Systematic therapy is the standard scheme for advanced HCC with vascular invasion. However, HCC with vascular invasion exhibits great heterogeneity in disease characteristics and prognosis, resulting in limited efficacy of systematic therapy 73. On the other hand, although ablation is a minimally invasive and effective modality for rHCC, some patients receiving ablation have a high risk of disease progression. Thus, new strategies are urgent needed for these patients.

It is reasonable to combine local treatment with systemic therapy (such as Vascular endothelial growth factor (VEGF) and multitarget tyrosine kinase inhibitors (TKIs)): 1. TKIs can kill and inhibit residual and micrometastatic lesions directly; 2. TKIs inhibit neovascularization induced by local treatment to prevent recurrence and metastasis; 3. TKIs improve the immunosuppressive effect in the tumor microenvironment and activate antitumor immunity. However, the STORM study showed that adjuvant sorafenib offers no significant survival benefit following surgical resection or ablation 74. Clinical trials in this field are ongoing, and the results have the potential to change the treatment strategy for rHCC 8.

Adjuvant sorafenib following RFA significantly improved OS and treatment free survival (TFS) compared with RFA alone. A quantitative risk score system was established to precisely identify the population that will benefit from RFA-sorafenib treatment. For rHCC patients within the Milan standard after the first hepatectomy, RFA-sorafenib significantly improved OS compared with RFA alone. Subgroup analyses concluded that patients with high risk scores had significantly longer survival after sorafenib administration 75.

Early rHCC patients with microvascular infiltration (MVI) at initial hepatectomy (tumor number ≤ 3 and tumor size 2-5 cm) received RFA treatment with poor effect. A retrospective study involving 211 patients found that adjuvant sorafenib following RFA was associated with better survival than RFA alone in patients with early-stage rHCC with MVI at the initial hepatectomy, and MVI grade could guide the application of adjuvant sorafenib 76.

Qi et al. found that sunitinib combined with RFA significantly inhibited tumor growth and prolonged survival. Further studies found that the combined treatment could significantly increase CD8+ T cells and dendritic cells in the tumor microenvironment, reduce inhibitory T cells, and activate the tumor-specific antigen immune response. Sunitinib can inhibit programmed death 1 (PD-1) expression in tumor-infiltrating T lymphocytes induced by RFA by inhibiting the hepatocyte growth factor (HGF) and VEGF signalling pathways. Sunitinib also inhibits the expression of programmed cell death 1 ligand 1 (PD-L1) on the surface of dendritic cells by inhibiting the VEGF effect, thereby attenuating the heat sink effect 77.

Immune checkpoint inhibitors (ICIs) can relieve the immunosuppressive state of effector T cells and enhance the immune killing effect of T cells on tumors 78. Although ICIs exhibit promising antitumor activity in clinical trials, they are effective in only a minority of patients. Compared with TKI monotherapy, combination therapy based on ICIs has become the first-line and new standard treatment for unresectable HCC 79,80.

At present, the mechanism of the antitumor effect of ablation combined with immunotherapy is unclear. It has been demonstrated that ablation leads to the release of a large amount of tumor-specific antigens, activating antigen-presenting cells. This process results in the subsequent activation of immune cells, including cytotoxic T cells and NK cells etc, to exert antitumor effects, and this effect not only effectively controls target lesions but also exerts inhibitory effects on distant metastatic lesions 81-85. In addition, thermal ablation induces various biological effects independent of tumor antigen release, including heat stress induced autophagy and the induction of proinflammatory cytokines, such as IL-1β, IL-6, IL-8, TNF, and HSP70 86-88. Therefore, the theoretical basis of ablation combined with immunotherapy may include the following: 1. ablation effectively reduces tumor load; 2. ablation induces the release of tumor-related antigens and cytokines and affects the antitumor immune response; 3. immunotherapy removes residual lesions and eliminates potential and distant metastatic lesions 89.

RFA combined with PD-1 antibody enhanced the tumor-specific immune response in a mouse model 90. IRE combined with PD-1 antibody promoted the infiltration of CD8+ T cells into tumors, significantly prolonging the survival of mice 91. Huang et al. demonstrated that MWA combined with PD-1 antibody treatment inhibited the growth of abscopal tumors, regulated the tumor microenvironment, improved the survival rate, and reduced recurrence by enhancing antitumor immunity 92.

A nonrandomized, phase 1/2, single-arm study found that ablation in combination with tremelimumab was a potential new treatment for patients with advanced HCC and led to the accumulation of intratumoral CD8+ T cells 93. Leuchte et al. analysed the relationship between the tumor-specific immune response, T-cell response in the peripheral blood and disease outcome of patients with HCC after thermal ablation. They reported that 30% of patients had a new or enhanced tumor-specific immune response, which was related to the efficacy of tumor ablation. The number of tumor-specific T cells was significantly related to PFS 94.

As a key molecule, VEGF inhibits the function of T cells, promotes the recruitment of regulatory T cells (Treg cells), myeloid-derived suppressor cells (MDSCs) and mast cells, and hinders dendritic cell differentiation and activation. Of note, VEGF can modulate the expression level of checkpoints on the surface of T cells 95. Targeting VEGF can enhance antitumor immunity and enhance the therapeutic effect of ICIs through various mechanisms, including normalizing tumor vasculature and enhancing T-cell infiltration 96-98.

TKIs switch so-called immune "cold" tumors to so-called "hot" tumors which are characterized by dendritic cell activation, T-cell infiltration, increased tumor antigen presentation and increased interferon signalling, by blocking MAPK, WNT-β-catenin, CDK4/6 or PTEN-dependent signalling. Thus, TKIs might enhance the antitumor effects and reduce drug resistance to ICIs 99,100. Li et al. reported that a patient with metastatic HCC who had disease recurrence following surgery and then received CRA followed by combined lenvatinib and toripalimab achieved a complete response after 7 months of treatment and a PFS of 24 months in the latest reported 101.

**Figure 2:**
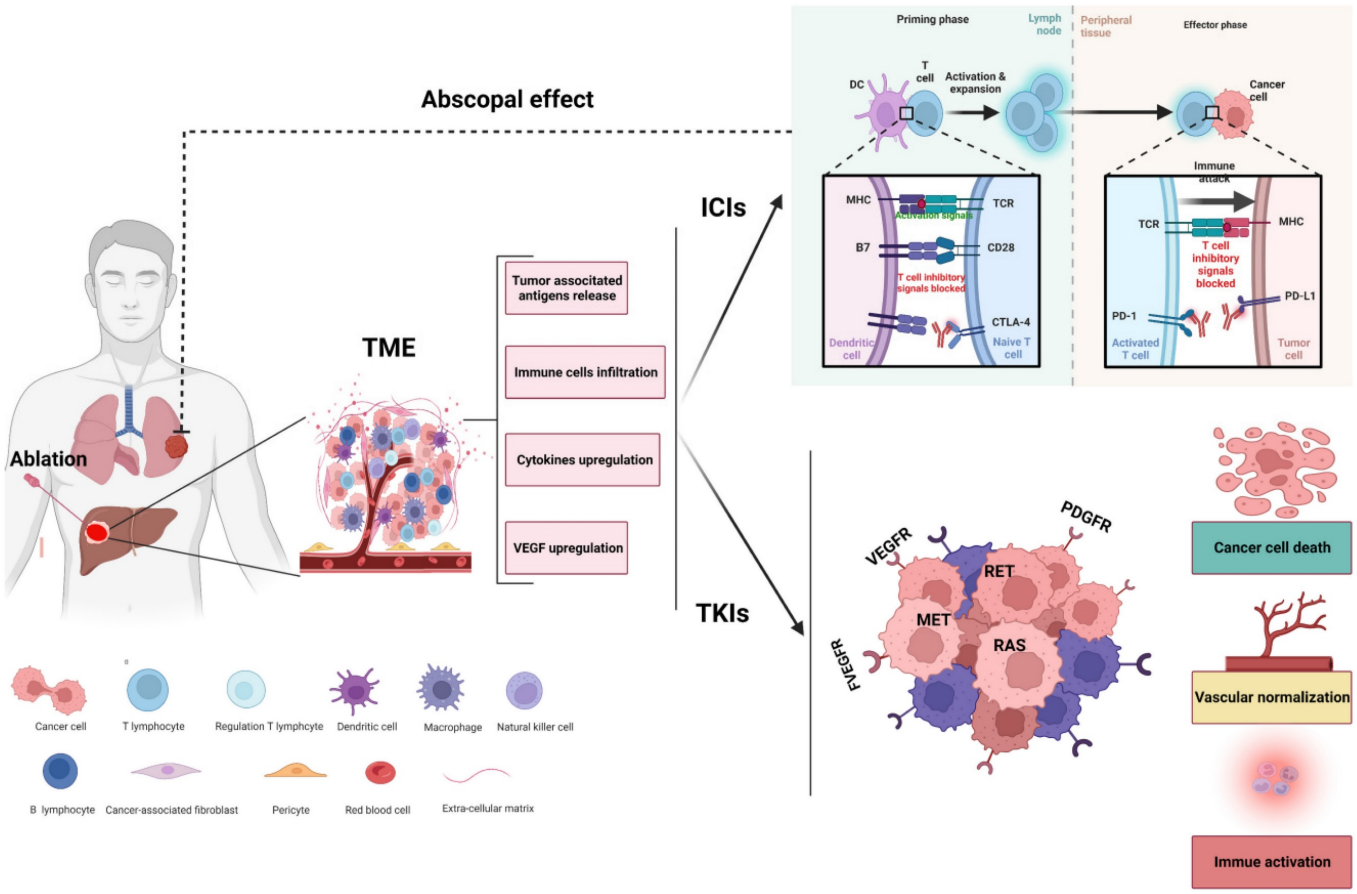
Rationale for ablation combined with systemic therapies. Ablation of HCC might shape tumor immunity by altering the composition of the tumor microenvironment (TME). Ablation leads to necrosis of tumor cells and induces the release of tumor-associated neoantigens and cytokines, facilitating the recruitment and activation of immune cells into the microenvironment and enhancing the antitumor effect of immune checkpoint inhibitors (ICIs). In addition, tyrosine kinase inhibitors (TKIs) can remodel the TME by killing tumor cells, normalizing the vasculature and transforming a nonimmunogenic 'cold' tumor into an inflamed 'hot' tumor. The synergistic effect of ablation combined with systemic therapies might offer increased efficacy not only for local tumors but also for abscopal lesions.

Although the combination of ablation and systematic treatment (including TKIs, VEGF targeting and ICIs) may represent an effective strategy for HCC, there are still many questions that need to be further investigated regarding the combination of ablation. For example, must tumor lesions be ablated completely? Is the best time point for immune combination before or after ablation? The underlying hypothesis of the synergistic effect of ablation combined with systemic therapies for the treatment of HCC is presented in Figure 2.

## Guidance techniques- Vital factor determining the effectiveness and safety of ablation

Precise puncture and placement of the ablation electrode can effectively reduce LTP. Currently, ultrasound (US), computer tomography (CT) and magnetic resonance (MR) can be used to guide ablation procedures. US can dynamically monitor the puncture process in real time and accurately puncture the lesions, especially for lesions located in special areas (such as the caudate lobe) 102. Although CT can better monitor the ablation effect immediately after surgery, CT is not real-time imaging, and puncture of relatively small lesions may be more challenging than US and radiation. Hermida et al. proposed that US should be the preferred guidance method for 2- to 3cm HCC lesions 103. MR is a safe and feasible MWA guidance and monitoring method that can reduce the incidence of LTP, especially when it is not suitable for CT or US guidance 104.

Li et al. compared the differences between MR and CT guided MWA and found that tumor diameter (<3 cm) and number of lesions (single) were important factors affecting LTP and OS. No significant difference in survival was noted between two groups. However, MR guided can reduce the incidence of complications 105.

Compared with US or CT guidance alone, US combined with CT significantly reduced the median number of punctures, LTP and serious complications. Therefore, the combination of US and CT guided ablation is better than US or CT alone 106.

Fusion imaging improves the effectiveness and safety of thermal ablation, which may be better for large lesions or lesions at special sites 107. US/CT-MR fusion imaging provides better visualization of the tumor and improves the success rate of ablation, and multi-needle ablation under the guidance of fusion imaging technology is associated with better outcomes and lower local recurrence 108. US/MR fusion imaging-guided RFA can be used to precisely ablate 5. 5-10 mm of rHCC 109.

Novel navigation system provides more precise and clearer guidance for the ablation of HCC lesions in special locations, such as sub-diaphragmatic lesions 110. For lesions in the caudate lobe, 3D visualization-assisted US-guided MWA is less expensive and has a shorter hospital stay than surgical resection 111. For lesions with a diameter greater than 3 cm, 3D visualization-assisted US-guided MWA has a lower local recurrence rate 112.

## Anaesthesia methods- General anaesthesia is recommended

Ablation therapy is generally performed under moderate sedation or general anaesthesia (GA), and it should be noted that patient assessment for procedural sedation risk using various methods such as the American Association of Anaesthesiologists (ASA) classification score is critical 20.

Percutaneous MR-guided MWA is generally performed under local anaesthesia (LA) and sedation traditionally, but the pain is difficult to control in these cases, especially in certain circumstances, such as when the lesion is large or located in a specific location (e.g., adjacent to the abdominal wall or diaphragm). A retrospective study compared the difference between GA and LA in patients undergoing MR-guided MWA for HCC. The ablation procedure was successfully completed in all patients, and a significant difference in the average ablation time was noted between the GA and LA group. No significant difference in complications or LTP were noted between the two groups. Notably, tumor location (specific location) and number of lesions (2-3 lesions) potentially represent the main factors affecting LTP. Univariate Cox proportional hazard regression showed that the use of different anaesthesia methods was not associated with LTP, whereas both tumor location (challenging location) and number of lesions (2-3 lesions) were associated with shorter LTP. In addition, multivariate Cox regression further showed that tumor location (regular location) and the number of lesions (1 lesion) independently predicted better LTP. For patients undergoing MR-guided MWA, the incidence of LTP was not correlated with anaesthesia modality 113.

For patients with HCC lesions at special locations, GA is superior to LA plus intraoperative analgesia when undergoing ablation, and GA can reduce the difficulty of the procedure and improve the safety of ablation 114.

## Efficacy evaluation scheme- Multifactorial predictive models based on artificial intelligence are increasingly used

As the 5-year recurrence rate of ablation for HCC is approximately 70-80%, and systemic therapy (including targeted therapy and ICIs, etc.) has a significant impact on the outcome, it is crucial to evaluate the efficacy of ablation 76,115,116.

Criteria for evaluating the effectiveness of ablation include LTP, RFS, DFS and OS etc generally. Moreover, baseline tumor biomarker (such as AFP) levels are helpful to monitor the response to ablation. MVI status plays an important role in the selection of treatment strategies for HBV+ rHCC patients. For MVI (-) patients, the prognosis of ablation is better than that of TACE. However, for MVI (+) patients, no significant differences in prognosis are noted in patients subject to ablation versus TACE 117.

Investigations based on multifactorial predictive models with artificial intelligence are increasingly used. A study that included 238 patients undergoing ablative therapy for HCC found that several factors, such as tumor size and AFP, were associated with ablation efficacy 110. Another study found that a selection model with 5 different characteristics predicted the outcome of ablative therapy for HCC patients 118. Among 252 patients undergoing ablation, the artificial neural network model based on 15 clinical variables can effectively predict DFS at 1 and 2 years 119. The nomogram model can accurately predict local recurrence in patients undergoing ablation and assist clinicians in making treatment decisions 120.

An et al. reported that three machine learning models (random forest, support vector machine, and eXtreme Gradient Boosting (XGBoost)) were superior to traditional logistic registration in predicting early recurrence after ablation. The XGBoost model was the best predictor when 9 variables (tumor number, platelet count, α-fetoprotein, comorbidity score, white blood cell count, cholinesterase, prothrombin time, neutrophils, and aetiology) were extracted simultaneously using recursive feature elimination and cross-validation. The XGBoost based hierarchical prediction system is available online (https://xgboost.readthedocs.io/en/stable/) 121.

## Complications- Different ablation modalities exhibit similar incidences of major complications

Common complications of thermal ablation include bleeding, visceral perforation, abnormal liver function, biliary tract injury, pneumothorax, and skin burns 13,20. CRA and thermal ablation exhibit similar incidences of major complications 20. However, a significant complication of liver CRA is a complex systemic inflammatory response called "cryo-shock", which includes multisystem organ failure, haemodynamic compromise, thrombocytopenia, and diffuse intravascular coagulation 122. In addition to adverse events, such as bleeding and liver function abnormalities, a history of ventricular arrhythmias is a relative contraindication to IRE because the strong current transmitted during IRE can trigger haemodynamic changes and lead to ventricular arrhythmia 57.

## Future prospects

rHCC could benefit from deeper insights into long-term outcomes and inclusion of newer clinical data, to further enrich its relevance in the rapidly evolving field of rHCC treatment. In the future, efforts can be made to improve the effectiveness and safety of ablation in HCC, including but not limited to: 1. investigating novel ablation procedures and exploring combination pattern with other anti-tumor schemes; 2. develop strategies to reduce adverse reactions and complications based on the characteristics of different ablation modalities; 3. identifying biomarkers and establishing analytical models to screen the potential patients who can benefit from ablation, and on the other hand, to predict efficacy and prognosis of ablation treatment.

In addition, an important aspect of evaluating HCC treatment is how to better understand and layout local and systemic strategies to achieve personalized and precise treatment, while addressing these issues requires in-depth research and understanding of the biological behavior and clinical characteristics of HCC.

## Abbreviations

AUC: area under the curve
LS: least squares
rHCC: recurrent hepatocellular carcinoma
RFA: radiofrequency ablation
MWA: microwave ablation
LA: laser ablation
CRA: cryoablation
HIFU: high-intensity focused ultrasound
IRE: irreversible electroporation
HCC: hepatocellular carcinomas
NCCN: national comprehensive cancer network
ECOG: eastern Cooperative Oncology Group
BCLC: barcelona clinical liver cancer
LRT: loco-regional therapy
IRE: irreversible electroporation
TACE: transarterial chemoembolization
PEI: percutaneous ethanol injection
SEER: surveillance epidemiology and end results
mOS: median overall survival
mCSS: median cancer-specific survival
PSM: propensity score matching
AJCC: American joint committee on cancer
LTP: local tumor progression
RHR: hepeat surgical resection
OS: overall survival
DFS: disease-free survival
PFS: progression-free survival
METTL1: Methyltransferase 1
CA: complete ablation
LR: local recurrence
nsPEF: nanosecond pulsed electric field
LRF: local recurrence-free
CTN: complete tumor necrosis
VEGF: vascular endothelial growth factor
TKIs: tyrosine kinase inhibitors
TFS: treatment-free survival
MVI: microvascular infiltration
HGF: hepatocyte growth factor
PD-1: programmed death 1
PD-L1: programmed cell death ligand 1
ICIs: immune checkpoint inhibitors
Treg cells: regulatory T cells
MDSCs: myeloid-derived suppressor
US: ultrasound
CT: computer tomography
MR: magnetic resonance
GA: general anaesthesia
ASA: American association of anaesthesiologists
LA: local anaesthesia
XGBoost: extreme gradient boosting
TME: tumor microenvironment
